# Non-ossifying fibroma with a pathologic fracture in a 12-year-old girl with tricho-rhino-phalangeal syndrome: a case report

**DOI:** 10.1186/s12881-018-0732-4

**Published:** 2018-12-12

**Authors:** Weijuan Su, Xiulin Shi, Mingzhu Lin, Caoxin Huang, Liying Wang, Haiqu Song, Yanzhen Zhuang, Haifang Zhang, Nanzhu Li, Xuejun Li

**Affiliations:** 1 Department of Endocrinology and Diabetes, The First Affiliated Hospital of Xiamen University, 55# Zhenhai Road, Xiamen, 361003 China; 2 Xiamen Diabetes Institute, the First Affiliated Hospital of Xiamen University, Xiamen, 361003 China; 3 Department of Pathology, the First Affiliated Hospital of Xiamen University, Xiamen, 361003 China; 4 Department of Pediatric Orthopaedics, the First Affiliated Hospital of Xiamen University, Xiamen, 361003 China

**Keywords:** Non-ossifying fibroma, Tricho-rhino-phalangeal syndrome, Fibula fracture, TRPS1 gene

## Abstract

**Background:**

Tricho-rhino-phalangeal syndrome (TRPS) is a rare autosomal dominant genetic disorder characterized by distinctive craniofacial and skeletal abnormalities, while non-ossifying fibroma (NOF) is a common benign bone tumour in children and adolescents. To date, no case of TRPS coexisting with NOF has been reported. This report presents a 12-year-old girl who had the characteristic features of tricho-rhino-phalangeal syndrome and non-ossifying fibroma with a fibula fracture.

**Case presentation:**

A 12-year-old girl was admitted to the Department of Endocrinology and Diabetes for evaluation of brachydactyly and a right fibula fracture. Clinical examination revealed sparse scalp hair, a characteristic bulbous pear-shaped nose, and brachydactyly with significant shortening of the fourth metatarsal. Neither intellectual disability nor multiple exostoses were observed. Radiography of both hands showed brachydactyly and cone-shaped epiphyses of the middle phalanges of the digits of both hands with deviation of the phalangeal axis. Genetic analysis of *TRPS1* identified a heterozygous germline sequence variant (p.Ala932Thr) in exon 6 in the girl and her father. Approximately 1 month before being admitted to our department, the girl experienced a minor fall and suffered a fracture of the proximal fibula in the right lower limb. The pathological cytological diagnosis of the osteolytic lesion was NOF. Ten months following the surgery, the lesion on the proximal fibula of the girl disappeared.

**Conclusions:**

In conclusion, the present study is the first to report a rare case of NOF with a pathologic fracture in the fibula of a girl with TRPS. The identification of a missense mutation, (p.Ala932Thr), in exon 6 of *TRPS1* in this kindred further suggested that the patient had type I TRPS and indicated that mutations in this exon may be correlated with more pronounced features of the syndrome. Radiological techniques and genetic analysis played key roles in the definitive diagnosis.

**Electronic supplementary material:**

The online version of this article (10.1186/s12881-018-0732-4) contains supplementary material, which is available to authorized users.

## Background

Tricho-rhino-phalangeal syndrome (TRPS) is a rare autosomal dominant genetic disorder characterized by distinctive craniofacial and skeletal abnormalities [1]. TRPS patients generally present distinctive faces with sparse and slow-growing scalp hair, large protruding ears, laterally sparse eyebrows, a bulbous pear-shaped nose, a thin upper lip, an elongated philtrum, and bone abnormalities, including mild to severe brachydactyly, cone-shaped epiphyses, hip dysplasia and short stature [1–5]. The *TRPS1* gene was identified and mapped to chromosomal band 8q24.1 by Momeni et al. in 2000 [6]. TRPS can be further distinguished into type I and type II according to clinical characteristics and molecular changes. TRPS I (OMIM 190350), the classical type of TRPS, occurs as a consequence of missense mutations or chromosomal abnormalities in the *TRPS1* gene [2, 7, 8]. TRPS II (OMIM 150230), known as Langer-Giedion syndrome, is distinguished from TRPS I by intellectual disability and multiple exostoses, and it is considered a contiguous genetic syndrome caused by heterozygous deletions in 8q23.3-q24.11 that involve both the *TRPS1* and *EXT1* genes [9].

Non-ossifying fibroma (NOF) is a benign, non-neoplastic lesion histologically characterized by a benign fibroblastic proliferation admixed with osteoclast-type giant cells [10]. NOF is most frequently observed in the metaphyseal region of the distal femur and in the proximal and distal tibia in children and adolescents, while its occurrence in the fibula is less common, at approximately 9.09% [11]. To our knowledge, no case of TRPS coexisting with NOF has been reported. Herein, we describe a case of NOF with a pathologic fracture in the fibula of a girl with TRPS I and carrying a missense mutation, (p.Ala932Thr), in exon 6 of *TRPS.*

## Case presentation

A 12-year-old girl was admitted to the Department of Endocrinology and Diabetes for evaluation of her brachydactyly and right fibular fracture. She was born after a full-term pregnancy and normal delivery with an Apgar score of 10 as the only child in a non-consanguineous Chinese family. No prenatal investigation was performed, and the motor development was normal. Upon birth, her fingers and toes were significantly stubby with obvious shortening of the fourth metatarsal, although neither hyperdactylia nor syndactylism was observed. No intellectual impairment or multiple exostoses were noticed. Pubertal development was normal. She experienced a minor fall 1 month before being admitted to our department, and she was diagnosed with a fracture of the upper fibula in the right lower limb, which was treated by fibula internal fixation and fibula bone grafting in the Department of Pediatric Orthopedics. To investigate the reason for the brachydactyly, the patient was referred to the Department of Endocrinology and Diabetes.

**Fig. 1:**
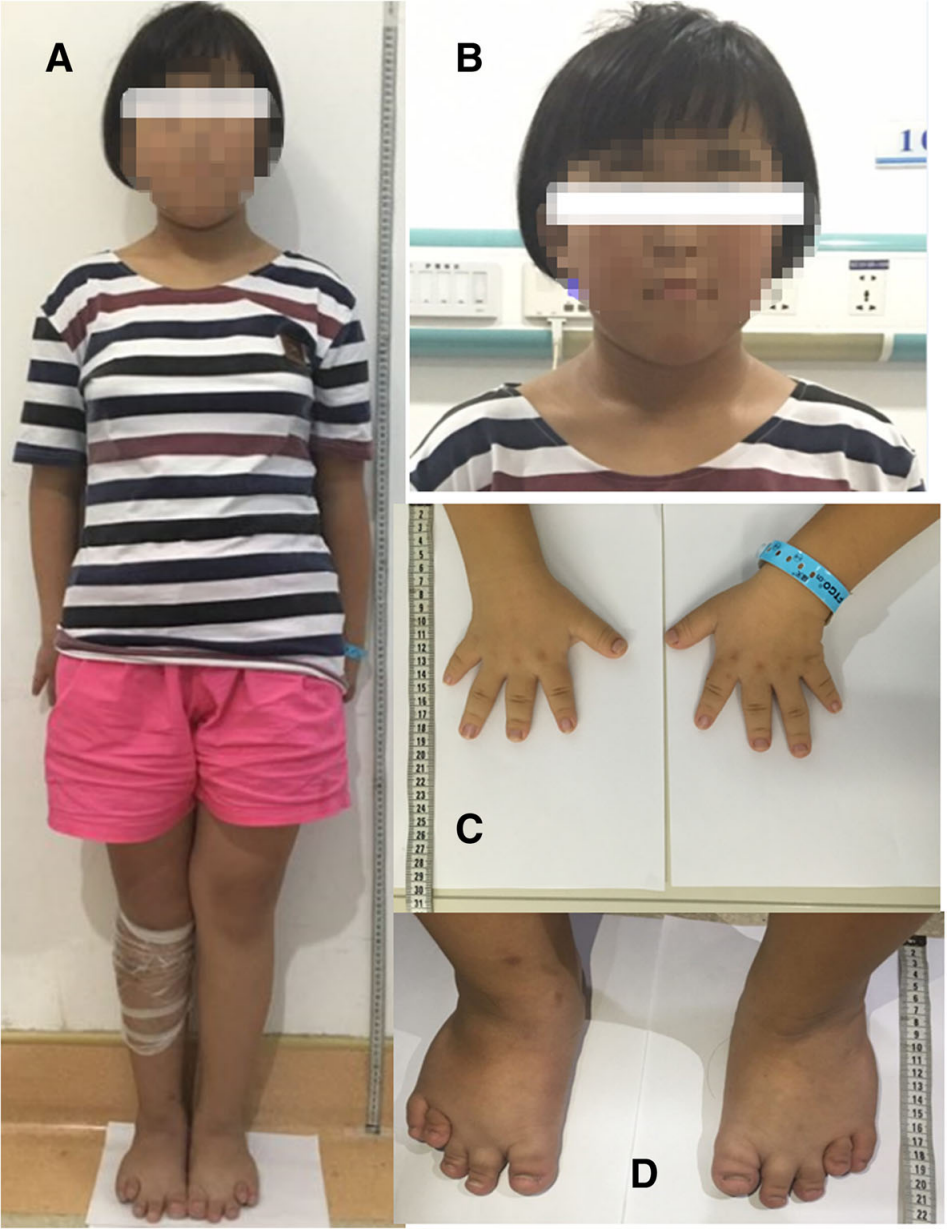
Typical dysmorphological features of the proband including short stature (**a**), pronounced facial characteristics (**b**) and shortening of the hands (**c**) and feet (**d**)

**Fig. 2:**
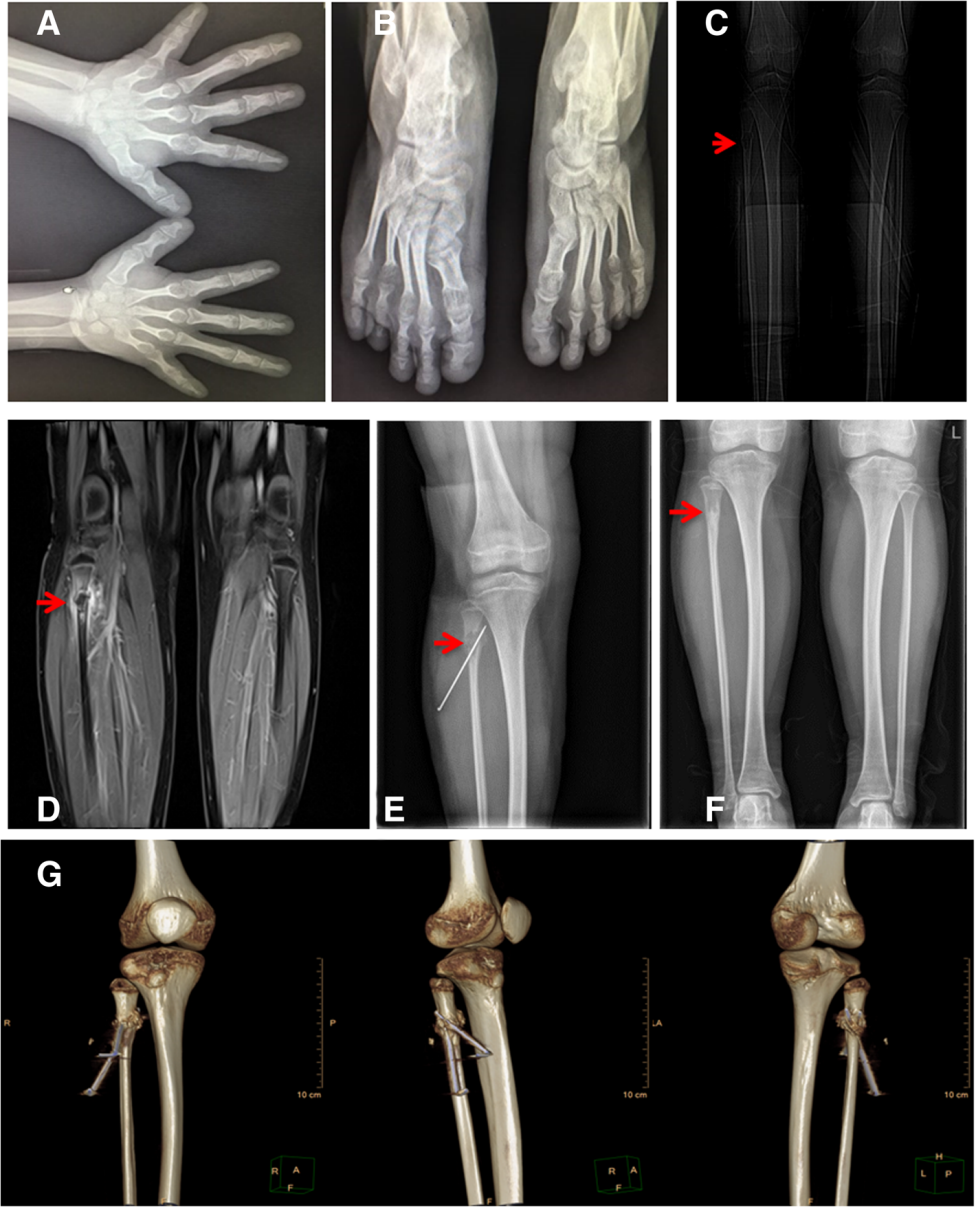
Radiography of the girl. **a**: Hands; **b**: Feet; **c**: Tibiofibular X-ray before the surgery; **d**: Fibula MRI; **e**: Tibiofibular X-ray 1 month after surgery; **f**: Tibiofibular X-ray 10 months after surgery; **g**: Tibiofibular three-dimensional CT 1 month after surgery

Upon admission, a routine clinical examination revealed that the patient’s standing height was 144 cm, with her upper portion measuring 72 cm and her lower portion measuring 72 cm, and her arm span was 131.3 cm (Fig. 1a). Clinical examination also revealed sparse scalp hair, a high-bossed forehead, thick eyebrows with lateral rarefaction, a characteristic bulbous pear-shaped nose, a long philtrum and a thin upper lip, pointed chin, and large, protruding ears (Fig. 1b). Examination of the extremities showed brachydactyly with significant shortening of the fourth metatarsal, flat feet, thin nails and koilonychias (Fig. 1c, d). Radiography of both hands (Fig. 2a) showed brachydactyly and cone-shaped epiphyses of the middle phalanges of the digits of both hands with deviation of the phalangeal axis. Similarly, cone-shaped epiphyses were also spotted in the proximal phalanx of the toes of both feet (Fig. 2b).

**Fig. 3:**
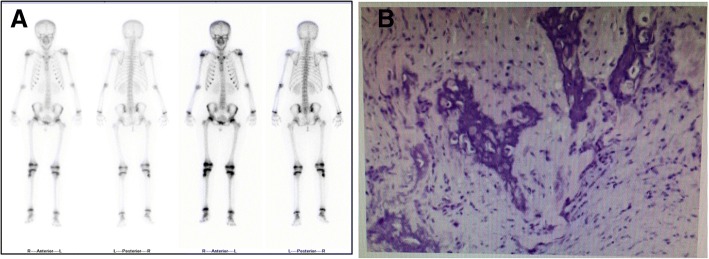
Whole-body bone scan and pathological cytology. **a**: Whole-body bone scan showing increased focal uptake at osteolytic lesion of the proximal right fibula; **b**: Pathological cytological examination showing the osteolytic lesion

Radiography was performed, and an osteolytic lesion was observed in the proximal right fibula before surgery. Radiography was performed 1 and 10 months following the surgery to monitor the recovery of the osteolytic lesion (Fig. 2c-g). Prior to the surgery, a large lesion protruding into the medullary cavity was found in the proximal right fibula with a pathologic fracture. A whole-body bone scan showed increased focal uptake only at the osteolytic lesion of the proximal right fibula (Fig. 3a).

A pathological cytological examination of the osteolytic lesion revealed that it was composed of benign spindle-shaped cells (fibroblasts) and histiocytes, with a scattering of xanthoma cells (Fig. 3b). Laboratory tests showed that the serum levels of calcium, inorganic phosphate, alkaline phosphatase, free T4, TSH, PTH, GH and IGF-1 were all normal.

**Fig. 4:**
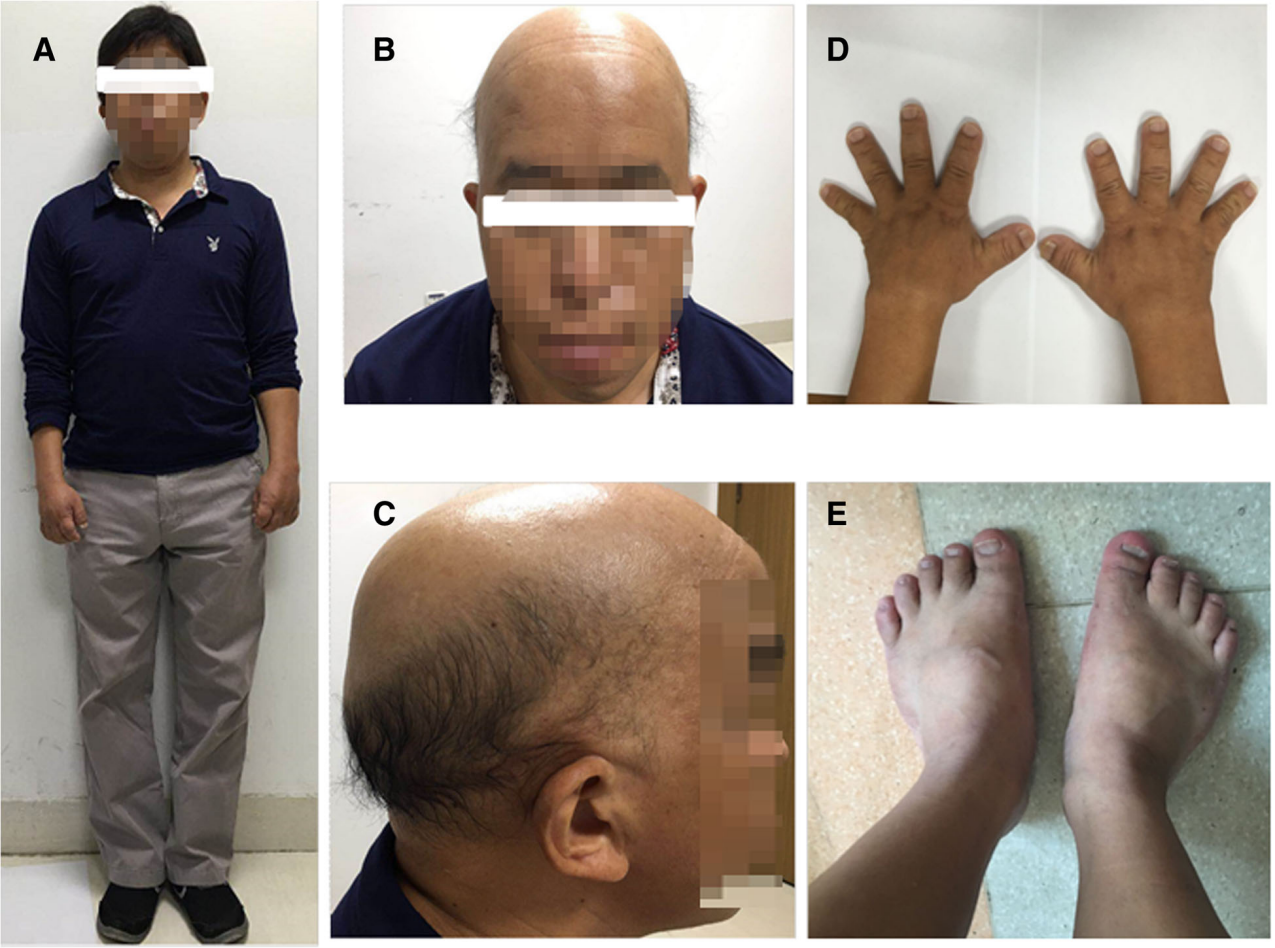
Typical dysmorphological features of the girl’s father including short stature (**a**), pronounced facial characteristics (**b**), diffuse alopecia (**c**) and shortening of hands (**d**) and feet (**e**). (The father wore a wig, as shown in Fig. 4 **a**)

The proband’s father presented with short stature (160 cm) (Fig. 4a), diffuse alopecia with fine hair, absence of lateral eyebrows, a large beaked nose and a long philtrum with a thin upper lip (Fig. 4b, c). He also presented with short metacarpals with brachydactyly, axial deviation of the middle finger and racket nails (Fig. 4d). No radiological examination was performed. The father declined radiological examination of his extremities.

**Fig. 5:**
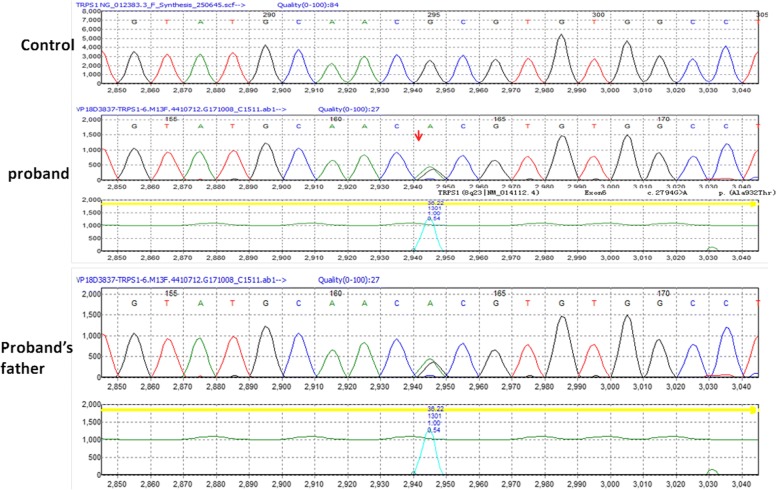
Gene sequencing of the girl and her father

Genetic analysis was performed using blood samples collected from the girl and her parents. A heterozygous variant, p.Ala932Thr, was identified in exon 6 of the *TRPS1* gene in the girl (Fig. 5). Subsequent targeted mutation analysis of exon 6 of her father confirmed the segregation of the variant in the girl. The healthy mother did not carry the sequence variant. Timeline depicting key milestones of diagnosis process and follow-up information was provided in the Additional file 1.

## Discussion and conclusions

TRPS is an autosomal dominant syndrome with high penetrance and wide phenotypic variability [12]. TRPS is characterized by sparse and slow-growing scalp hair, laterally sparse eyebrows, a bulbous pear-shaped nose, an elongated philtrum, and a thin upper lip. Furthermore, patients present with cone-shaped epiphyses and severe generalized shortening of all of their phalanges, metacarpals and metatarsal bones [13, 14].

The *TRPS1* gene, which encodes a transcription factor composed of 1294 amino acids encoded in seven exons with nine putative zinc-finger motifs, was identified by Momeni et al. in 2000 [6]. This protein is thought to function as a homodimeric nuclear regulator by interacting with GATA binding protein sequences in the DNA to repress transcription of its target genes, which themselves regulate chondrocyte proliferation, differentiation, and hair follicle proliferation [15]. Consistent with the clinical features of TRPS1, *TRPS1* is highly expressed in the affected organs of the patients, including the cartilage, developing joints, hair follicles and the nasal region [16, 17]. In this case, a missense mutation in codon 932 (p.Ala932Thr) was identified, in which a nonpolar alanine residue was replaced by threonine. Maas et al. performed a collaborative international study to delineate the phenotypic features, natural history, variability, and genotypic correlations of TRPS in more detail. They gathered information from 103 individuals in which TRPS was confirmed cytogenetically or molecularly, and they identified the same missense mutation in codon 932 (p.Ala932Thr) in 2 index patients [5]. Furthermore, two other missense mutations in the same codon, p.Ala932Ser and p.Ala932Val, have also been described as pathogenic mutations [5, 18].

Although TRPS is commonly classified into types I and II based on intellectual disability and multiple exostoses, another subtype of TRPS, TRPS III (OMIM 190351), has been referred to in earlier studies; TRPS III patients exhibit overlapping but more pronounced clinical manifestations of TRPS I, and they share the same molecular status as TRPS I patients but without the exostoses or intellectual disability associated with TRPS II [4, 19]. Thus, it is now suggested that TRPS III represents an extreme form of the clinical spectrum of TRPS I. Previous genotype-phenotype studies have connected missense mutations in exon 6 of *TRPS1* with TRPS III (which can be defined as a severe form of TRPS I), in which the patients present pronounced facial characteristics, short stature and brachydactyly, but who differ from type II patients due to the absence of exostoses and intellectual disability [5, 18, 20]. Consistently, some studies have shown that patients with missense mutations in exon 6 presented more pronounced facial characteristics and shortening of the hands and feet compared to patients with mutations in other exons [2, 5].

In our study, the girl and her father presented the typical characteristics of TRPS, including distinctive craniofacial and skeletal abnormalities, a bulbous nasal tip, large, protruding ears, brachydactyly, and cone-shaped epiphyses. Based on these typical clinical and radiological features, the diagnosis of TRPS was made. Consistently, genetic analysis of *TRPS1* revealed that the girl and her father carried a heterozygous germline sequence variant (p.Ala932Thr) in exon 6. After clinical examination, no intellectual disability or multiple exostoses were observed, and based on these observations, this patient was diagnosed with TRPS I. Additional related physical symptoms reported in TRPS patients, such as endocrine disorders, were also not present in this family [1].

NOF is a common type of benign fibrous lesion that occurs in the metaphyseal region of the long bones in the lower extremities. NOF can be diagnosed based on plain radiographs [21], and it is estimated to be present in approximately 30% of young patients in their first or second decade of life. A lesion is usually self-limiting and disappears by the age of 20 to 25 years in most cases [22]. Nevertheless, there is a certain risk for pathological fractures. NOF was classified into 4 stages by Ritschl et al. [23]. In Stage A, the lesions are small and oval and adjacent to the growth plate; in Stage B, the lesions are located at variable distances from the growth plates with thin sclerotic borders exhibiting more polycyclic, grape-shaped borders; in Stage C, mineralisation tends to start in the shaft and proceeds towards the growth plates with increasing sclerosis; and in Stage D, the lesions show complete sclerosis. The study showed that patients with stage B lesions have an increased risk of suffering bone fractures. However, no fractures were found in stage A, C or D patients [24]. NOF lesions larger than 50% of the bone diameter are more likely to lead to fractures [25].

In our study, the girl experienced a minor fall and suffered a fracture of the proximal fibula in the right lower limb. The pathological cytological diagnosis was NOF. Radiography revealed that the girl had a stage B lesion that occupied more than 50% of the transverse diameter of the fibula. Ten months following the surgery, the lesion in the proximal fibula disappeared. The slender anatomical structure of the fibula could have also contributed to the increased risk of suffering a pathological fracture.

The definitive aetiology of NOF is unclear, and NOF is considered to be a growth disturbance or dystrophic calcification rather than a tumour or neoplasm [26]. The girl carried a missense mutation in the *TRPS1* gene that affected the function of the gene product. Studies have shown that TRPS1 is involved in bone formation and mineralization. Endochondral ossification is a multi-step process that starts with mesenchymal condensation. TRPS1 performs multiple functions in proliferating chondrocytes, including regulation of their proliferation and apoptosis; furthermore, TRPS1 promotes the differentiation of proliferative chondrocytes into hypertrophic chondrocytes and regulates mineralization and the formation of the bone matrix [27]. Further investigation is required to determine whether TRPS1 dysfunction contributes to NOF with pathologic fractures.

Management of TRPS is principally supportive. For our patient, measurement of her linear growth and routine development assessments were performed regularly [28]. In the treatment of NOF, follow-ups, including clinical surveys and imaging at six- to twelve-month intervals, can be considered in cases of larger stage B lesions until the lesions reach stage C [24]. Stage B lesions were found to have an increased risk of fracture [23]. In our study, the lesion on the proximal fibula of the girl disappeared 10 months after the surgery. However, the missense mutation in *TRPS1*, which contributes to low bone formation and mineralization, could increase the risk of fracture. Therefore, the girl was advised to avoid falls and to regularly undergo bone mineral density assessment.

In conclusion, the present study first reports a rare case of NOF with a pathologic fracture in the fibula of a girl with TRPS I. The identification of a missense mutation (p.Ala932Thr) in exon 6 of *TRPS1* in this kindred provided further indication that mutations in this exon may correlate to a more significant manifestation of the clinical features of the syndrome. Radiological techniques and genetic analysis played key roles in making the definitive diagnosis.

## Additional file

Additional file 1: Timeline. (DOCX 13 kb)

## Abbreviations

Ala: Alanine
GH: Growth hormone
IGF-1: Insulin-like growth factor 1
NOF: Non-ossifying fibroma
PTH: Parathyroid hormone
Ser: Serine
T4: Thyroxine
Thr: Threonine
TRPS: Tricho-rhino-phalangeal syndrome
TSH: Thyroid stimulating hormone
Val: Valine
