# Supplementary material for: Non-ossifying fibroma with a pathologic fracture in a 12-year-old girl with tricho-rhino-phalangeal syndrome: a case report
Source: BMC Med Genet. 2018 Dec 12;19:211. doi: 10.1186/s12881-018-0732-4 (PMC6292130; doi:10.1186/s12881-018-0732-4)
Supplement: Supplementary file 1 — Timeline. (DOCX 13 kb) [file 12881_2018_732_MOESM1_ESM.docx]

**Timeline:** *“The Non-ossifying Fibroma with a pathologic fracture in a 12-year-old girl with Tricho-Rhino-Phalangeal Syndrome: a Case report”*

| **Time** | **Event** |
| --- | --- |
| **February 2005** | The patient was born and her fingers and toes were significantly stubby with obviously shortening of the fourth toes. Neither hyperdactylia or syndactylism was observed. |
| **June 4^th^, 2017** | She experienced a minor fall and suffered a fracture of the proximal fibula in right lower limb. |
| **June 10^th^, 2017** | Her fracture was treated by fibula internal fixation and fibula bone grafting in the Department of Orthopedics in children. |
| **June 17^th^, 2017** | The osteolytic lesion was diagnosed as The Non-ossifying Fibroma (NOF) by pathological cytological diagnosis. |
| **July 7^th^,2017** | She was admitted to the Department of Endocrinology and Diabetes for evaluation of brachydactyly and right fibula fracture. |
| **August 6^th^, 2017** | Gene analysis of *TRPS1* revealed that a heterozygous germline sequence variant (p.Ala932Thr) in exon 6 was identified in the girl and her father. |
| **April 12^th^, 2018** | 10 months following the surgery, lesion of the proximal fibula of the girl disappeared. |
